# Durable Disease Control in Disseminated Primary Splenic Leiomyosarcoma Treated With Sequential Gemcitabine–Docetaxel and Pazopanib Therapy: A Case Report

**DOI:** 10.7759/cureus.106553

**Published:** 2026-04-06

**Authors:** Saida Kurbanova, Biloliddin Sharobiddinov, Oqila Abdusattorova, Ravshan Abdusattorov

**Affiliations:** 1 Department of Oncology, AKFA Medline University Hospital, Tashkent, UZB; 2 School of Medicine, Central Asian University, Tashkent, UZB

**Keywords:** case report, docetaxel, gemcitabine, leiomyosarcoma, pazopanib, primary splenic leiomyosarcoma, soft tissue sarcoma, targeted therapy

## Abstract

Primary splenic leiomyosarcoma (PSL) is an exceptionally rare malignancy, and therapeutic guidance is limited to individual case reports. We describe a patient with disseminated PSL who achieved prolonged disease control using a sequential strategy combining cytotoxic chemotherapy and tyrosine kinase inhibitor (TKI) prolonged therapy. A 52-year-old woman presented with abdominal discomfort in the preoperative period (prior to Day 1). Magnetic resonance imaging demonstrated multiple splenic lesions with splenomegaly. Splenectomy confirmed leiomyosarcoma (French Federation of Cancer Centers Sarcoma Group (FNCLCC) grade 2; Ki-67 20-25%) with smooth muscle immunophenotype (smooth muscle actin and h-caldesmon positive). Postoperative staging with positron emission tomography/computed tomography (PET/CT) revealed diffuse metastatic disease involving the peritoneum, pleura, pericardium, and liver. She received four cycles of gemcitabine-docetaxel with partial metabolic response, followed by pazopanib (800 mg daily) as maintenance. After radiographic relapse, gemcitabine-docetaxel was reintroduced for four additional cycles. Overall, the initial response was followed by seven months of stable disease on pazopanib; after re-induction, the final documented progression occurred around Day 540. This case suggests that a cytotoxic-to-TKI sequential strategy may be feasible in disseminated PSL and was associated with prolonged disease control in this patient. Given the rarity of PSL, systematic case reporting and, when feasible, molecular characterization may help refine future management.

## Introduction

Primary leiomyosarcoma (LMS) of the spleen is extraordinarily uncommon, and most of the available literature consists of single-patient reports [1-3]. Primary splenic leiomyosarcoma (PSL) is exceptionally rare and typically presents with nonspecific symptoms or incidental splenic lesions on imaging. Diagnosis usually depends on splenectomy with histopathologic confirmation and clinicoradiologic exclusion of another primary site. Because no disease-specific treatment standard exists for disseminated PSL, systemic therapy decisions are generally extrapolated from broader LMS and soft-tissue sarcoma (STS) practice. 

Disseminated disease at presentation is associated with poor outcomes in STS. Standard first-line options typically include anthracycline-based therapy; however, gemcitabine-based combinations are frequently used for LMS because of demonstrated activity in randomized and phase II studies [4-6]. Anti-angiogenic therapy with pazopanib is an approved option for previously treated advanced STS and is sometimes used as prolonged therapy in responders [7].

Here, we report a case of disseminated PSL with a partial metabolic response to gemcitabine-docetaxel, followed by prolonged disease control during pazopanib therapy, and subsequent re-induction with gemcitabine-docetaxel. We prepared the report in accordance with the CARE (CAse REport) guideline framework.

## Case presentation

Clinical history and initial evaluation

A 52-year-old woman presented with several weeks of abdominal discomfort and distention. Past medical history was unremarkable. No prior gynecologic malignancy was known from the available clinical history. The patient was not taking regular medications at presentation.

**Figure 1:**
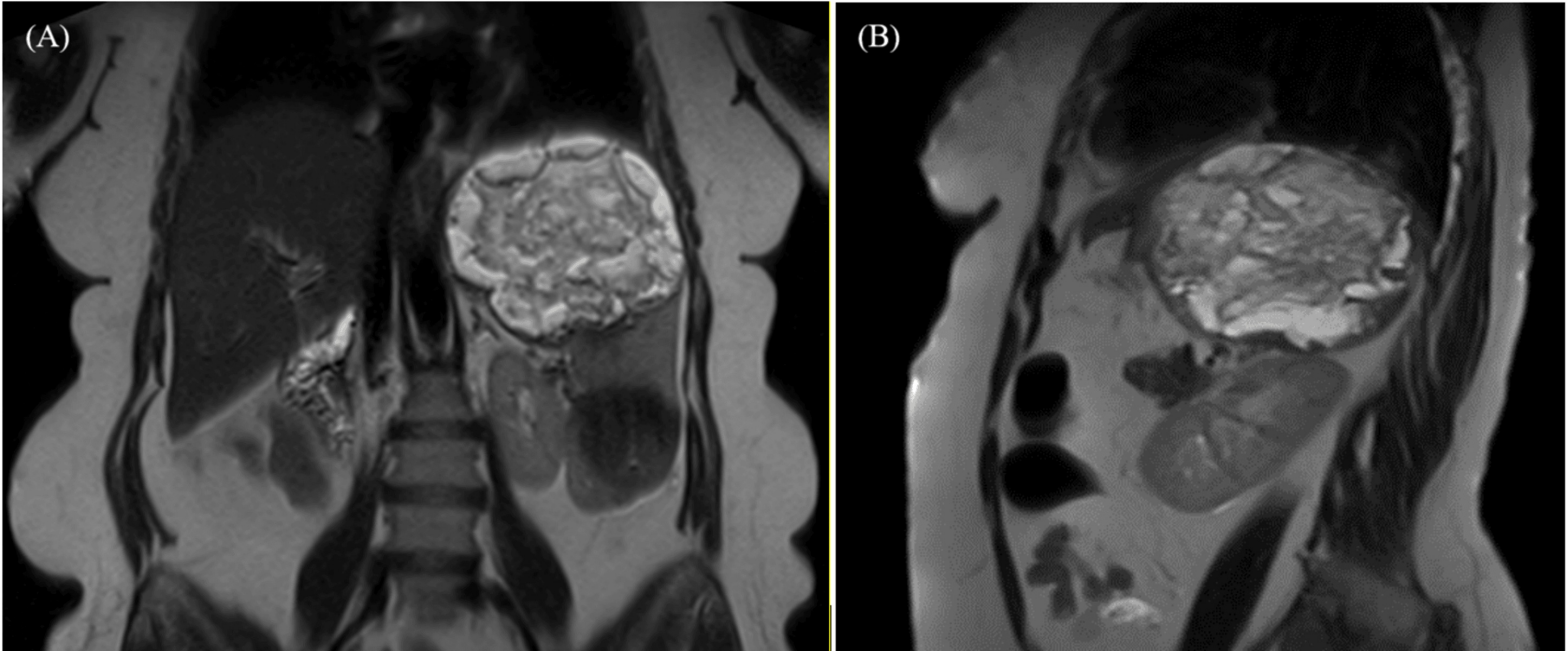
Preoperative abdominal MRI imaging. Coronal (A) and sagittal (B) MRI images reveal marked splenomegaly with mass-like formation.

Magnetic resonance imaging (MRI) of the abdomen (Figure 1) demonstrated splenomegaly with multiple intraparenchymal splenic lesions, initially suggestive of lymphoma. She underwent splenectomy (Day 1).

Pathological findings

**Figure 2:**
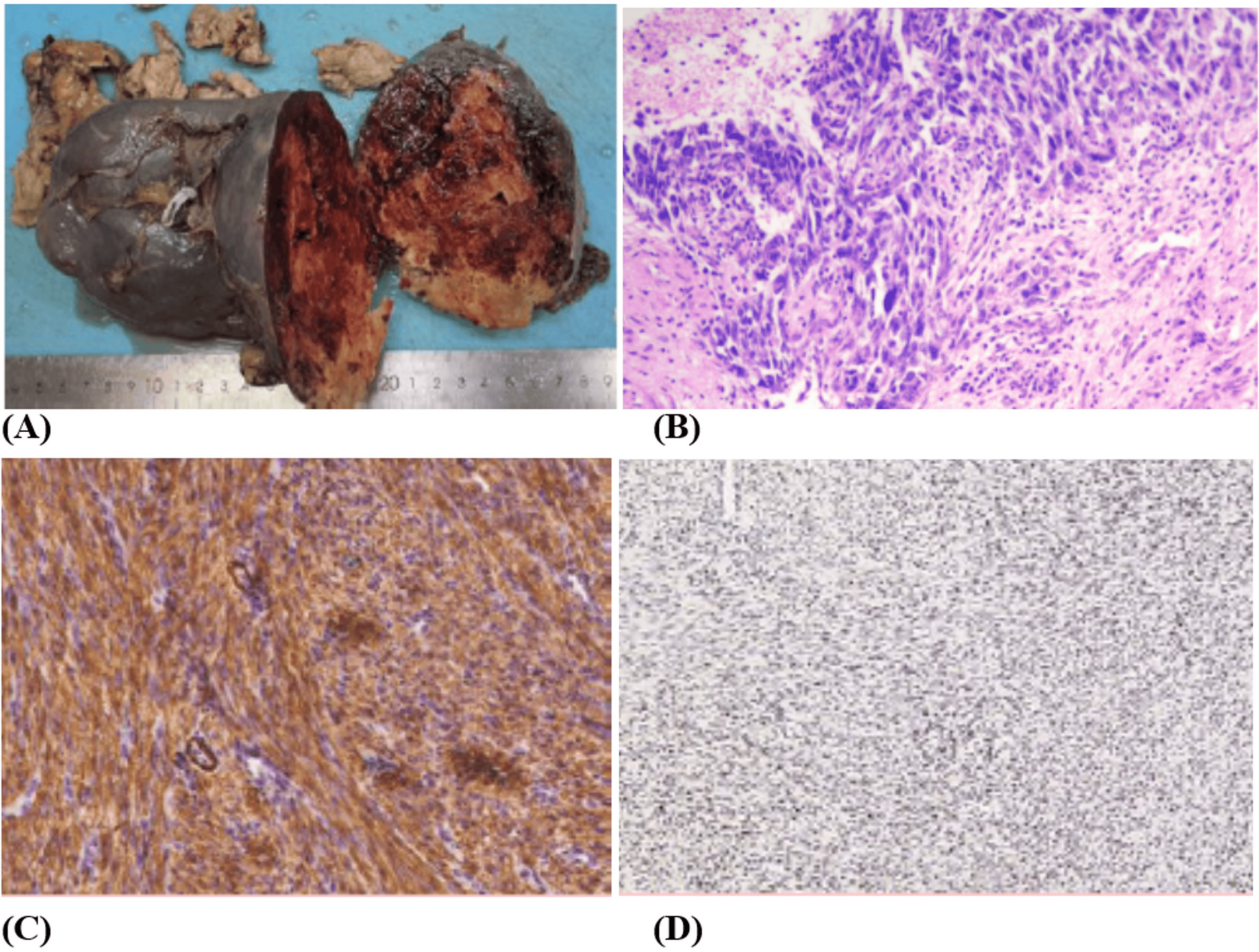
Gross pathology and histopathological findings. (A) Gross specimen of the spleen showing a 16 × 12 × 4.5 cm spleen with a light brown, soft tumor replacing approximately 20% of the parenchyma. (B) Hematoxylin and eosin staining demonstrating intersecting fascicles of spindle cells with eosinophilic cytoplasm, pleomorphic nuclei, and atypical mitoses (original magnification ×200). (C) Immunohistochemistry showing diffuse positivity for smooth muscle actin (SMA). (D) Immunohistochemistry showing positivity for h-caldesmon, confirming smooth muscle differentiation.

Gross examination revealed a spleen measuring 16 × 12 × 4.5 cm, with approximately 20% of the parenchyma replaced by a light brown, soft tumor measuring 4.5 × 1.2 × 0.8 cm with additional nodular areas ranging from 0.6 to 1.8 cm (Figure 2A). 

Histologically, the tumor consisted of intersecting fascicles of spindle-shaped cells with eosinophilic cytoplasm, pleomorphic nuclei, atypical mitoses, and necrosis, consistent with LMS (Figure 2B). Histopathologic examination showed LMS, FNCLCC grade 2 (total score 4: differentiation score 2; mitotic count score 1 (3/10 HPF); necrosis score 1 (20-25%)).

Immunohistochemistry supported smooth muscle differentiation, with positivity for smooth muscle actin (Figure 2C) and h-caldesmon (Figure 2D). The tumor was negative for CD45, BCL2, S100, CD117 (c-KIT), estrogen receptor, and progesterone receptor, thereby excluding key diagnostic mimics (e.g., lymphoma and gastrointestinal stromal tumor). Desmin, CD34, and DOG1 were not performed. The diagnosis, therefore, relied on the combination of morphology, the available immunophenotype, and clinicoradiologic correlation rather than immunohistochemistry alone.

Because LMS involving the spleen may represent metastatic disease from another occult primary site, attribution of splenic origin required clinicopathologic correlation. In this patient, the dominant splenic lesion was the presenting lesion, splenectomy pathology confirmed LMS with smooth muscle differentiation, and postoperative systemic imaging did not identify an alternative primary tumor. However, because a formal gynecologic examination was not performed, the primary splenic origin should be interpreted as the most likely clinicopathologic diagnosis rather than absolute proof of splenic origin.

Staging and treatment

Treatment response was assessed using serial CT/MRI and fluorodeoxyglucose (FDG) positron emission tomography/computed tomography (PET/CT). Formal RECIST 1.1 and PERCIST criteria were not prospectively applied because multifocal peritoneal and serosal disease limited reproducible target-lesion selection. The response was therefore described using radiologist-reported changes in lesion burden and trends in SUVmax over time. When multifocal peritoneal involvement limits reproducible anatomic measurements, FDG PET/CT may provide complementary information on treatment effect over time, although interpretation remains limited in diffuse peritoneal disease.

**Figure 3:**
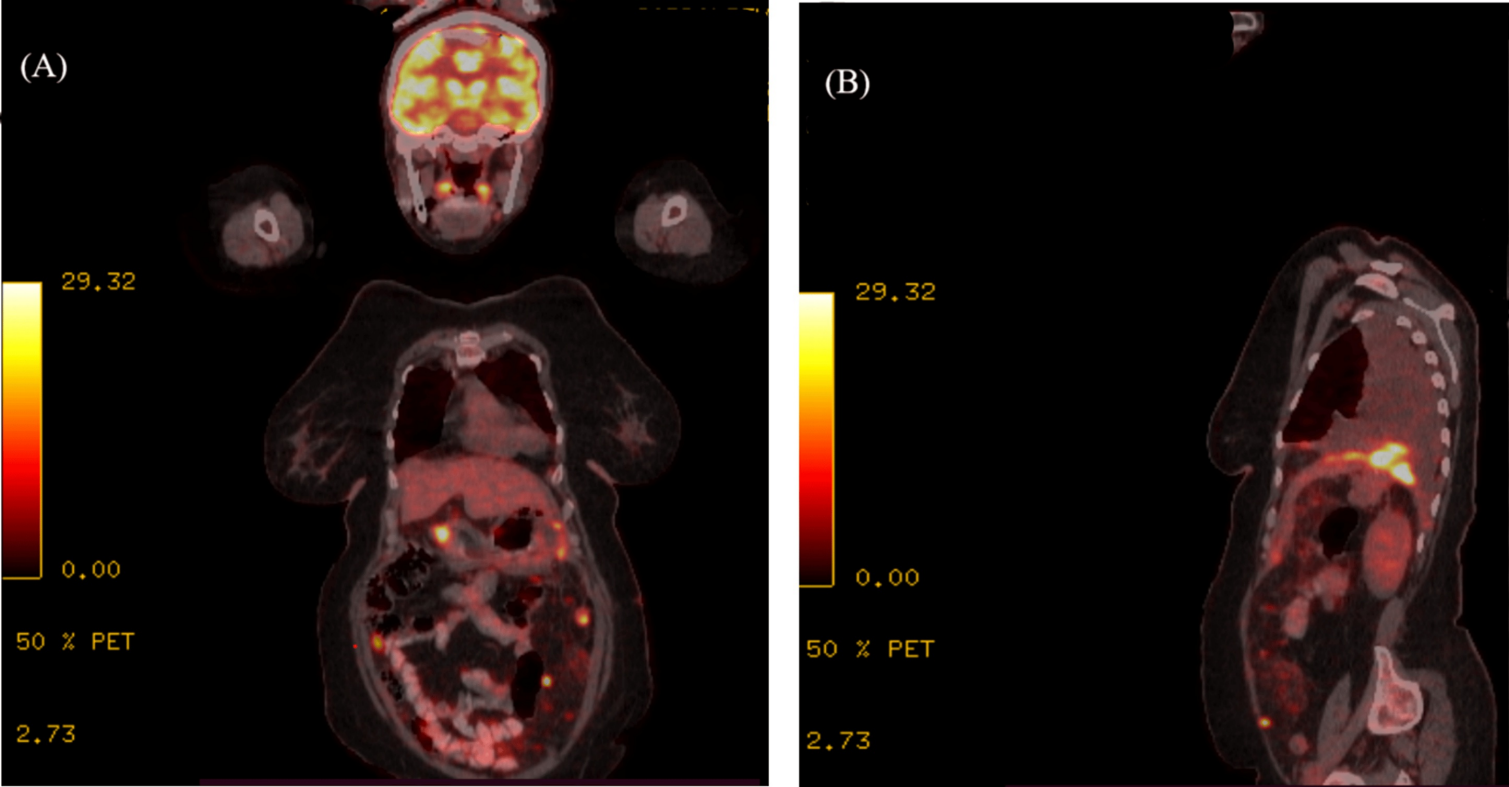
Representative imaging during treatment course. Initial staging PET/CT (Day 20-21) showing multiple metabolically active lesions in the peritoneum, pleura, and liver, in coronal (A) and sagittal (B) projections.

Postoperative staging included contrast-enhanced CT and FDG PET/CT on Days 20-21, which identified disseminated metastatic disease involving the peritoneum/mesentery, pleura/pericardium, and liver, accompanied by ascites (Figure 3). Given the extensive metastatic burden and the LMS histology, systemic therapy with gemcitabine and docetaxel was initiated.

Gemcitabine was administered at 1000 mg/m² on Days 1 and 8 and docetaxel at 75 mg/m² on Day 8 of a 21-day cycle (with supportive care per institutional practice). After four cycles, PET/CT on Day 124 demonstrated reduced FDG uptake and regression of previously described lesions, consistent with a partial metabolic response.

Based on the treatment response and the absence of PSL-specific systemic therapy standards, pazopanib 800 mg once daily was initiated on Day 169 as prolonged therapy. No clinically significant hematologic toxicity was documented during gemcitabine-docetaxel therapy; prophylactic filgrastim was administered after each cycle to reduce the risk of neutropenia. During pazopanib therapy, the patient experienced mild weakness and elevation of blood pressure up to 150 mmHg, which was controlled with a calcium-channel blocker. Because adverse events were not prospectively captured in a structured CTCAE format, detailed retrospective toxicity grading was not possible. Surveillance MRI on Day 308 showed stable residual disease.

Disease progression and subsequent therapy

**Figure 4:**
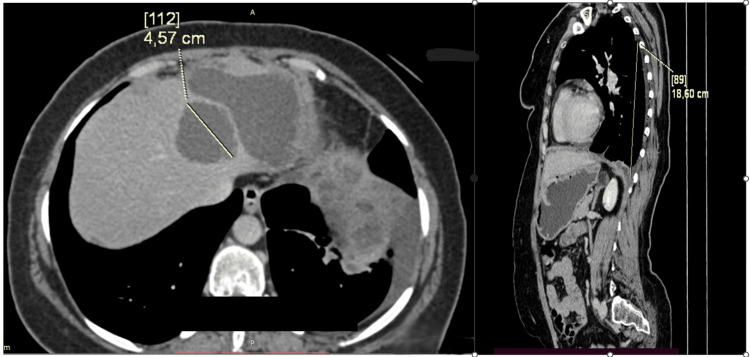
Representative imaging during the treatment course. Follow-up CT (Day 540) demonstrating radiographic progression of metastatic disease after sequential therapy. After initial response followed by approximately seven months of stable disease on pazopanib and subsequent re-induction, progressive disease was documented on Day 540.

On Day 399, CT demonstrated radiographic progression within the peritoneal compartment. Gemcitabine-docetaxel was reintroduced for four additional cycles (total of 8 across two treatment courses). On Day 540, CT demonstrated progressive disease (Figure 4). Subsequent-line therapy with trabectedin was initiated shortly after documented progression and continued through at least the time of manuscript preparation.

A summary of the clinical timeline is provided in Table 1.

**Table 1:** Patient timeline. SMA: Smooth muscle actin; FNCLCC: French Federation of Cancer Centers Sarcoma Group; FDG: fluorodeoxyglucose

Relative timepoint (Day)	Event	Details
Preoperative period (prior to Day 1)	Presentation	Presentation with abdominal discomfort and abdominal MRI showing splenomegaly with multiple intraparenchymal splenic lesions; initial differential included lymphoma.
Day 1	Splenectomy	Histopathology confirmed leiomyosarcoma (FNCLCC grade 2; Ki-67 ~20–25%) with SMA and h-caldesmon positivity
Day 20–21	Postoperative staging	CT and FDG PET/CT demonstrated disseminated metastatic disease (peritoneum/mesentery, pleura/pericardium, liver) with ascites.
Day ~22 to Day ~106	Systemic therapy (Course 1)	Gemcitabine–docetaxel, four cycles q21 days
Day 124	Response assessment	FDG PET/CT showed reduced FDG uptake and regression of lesions, consistent with partial metabolic response
Day 169	Prolonged therapy	Pazopanib 800 mg once daily initiated as prolonged therapy
Day 308	Follow-up imaging	Surveillance MRI showed stable residual disease
Day 399	Progression	CT demonstrated radiographic progression in the peritoneal compartment
Day ~400 to Day ~484	Systemic therapy (Course 2)	Gemcitabine–docetaxel re-induction, four additional cycles (eight cycles in total across both courses)
Day 540	Progression	Follow-up CT demonstrated progressive disease
After Day 540	Subsequent therapy	Trabectedin initiated; three cycles administered

## Discussion

This case illustrates several pragmatic lessons for clinicians facing an extremely rare primary splenic sarcoma.

First, PSL is so rare that treatment decisions are necessarily extrapolated from broader STS and LMS evidence. In metastatic STS, randomized data support the activity of gemcitabine-docetaxel compared with gemcitabine alone in pretreated populations, and the regimen is widely used in LMS, including uterine LMS [4-6]. In our patient, first-line gemcitabine-docetaxel produced a clear metabolic and clinical benefit.

Second, pazopanib, an oral multi-targeted tyrosine kinase inhibitor (TKI) with anti-angiogenic activity, was introduced after the initial chemotherapy response as prolonged therapy. In this patient, pazopanib provided a meaningful chemotherapy-free interval with stable residual disease before subsequent radiographic progression.

Third, tumor biology may be relevant even in individual cases. An intermediate proliferation index (Ki-67 20-25%) and FNCLCC grade 2 disease may partially explain the observed chemosensitivity and the ability to achieve disease control with sequential systemic therapies. However, robust biomarkers for chemotherapy response in LMS remain an unmet need.

Finally, this case highlights the potential value of molecular profiling in rare sarcomas. In this case, next-generation sequencing and targeted molecular testing were not performed due to financial constraints and limited affordability, with an estimated cost of approximately USD 2,500. Broader molecular testing may help refine diagnosis, exclude mimics, and, in selected cases, identify therapeutically relevant alterations; however, its utility in PSL remains uncertain.

Limitations

This report describes a single patient and cannot establish treatment efficacy. Molecular profiling was not performed, so no TMB, MSI status, or specific angiogenic pathway biomarkers were available. Detailed toxicity data were not systematically collected for reporting. Follow-up is ongoing as the patient is receiving subsequent-line therapy. Formal RECIST 1.1 and PERCIST criteria were not prospectively applied, and response assessment was based on pragmatic radiologist-reported imaging interpretation. Formal gynecologic examination was not performed, and primary splenic origin remains a diagnosis of exclusion. Adverse events were not prospectively captured in a structured CTCAE format, which limits the granularity of retrospective toxicity reporting. Next-generation sequencing was not performed due to financial constraints (estimated cost: approximately USD 2,500). Despite these limitations, the case contributes to the sparse literature on PSL and supports the feasibility of a cytotoxic-to-TKI sequence as a practical strategy to achieve meaningful disease control in disseminated disease.

Learning points

PSL is exceptionally rare, and attribution of splenic primary origin requires careful clinicopathologic exclusion of another occult primary site. In disseminated disease, a sequential strategy of cytotoxic chemotherapy followed by pazopanib may be feasible and provide a chemotherapy-free interval in selected patients, but this observation should be interpreted cautiously in a single case. When multifocal peritoneal disease limits reproducible anatomic measurements, FDG PET/CT may provide complementary information on treatment effect over time, although it does not replace standardized response criteria. In this case, intermediate-grade features (FNCLCC grade 2; Ki-67 approximately 20-25%) were observed alongside treatment responsiveness, but their predictive value for chemotherapy sensitivity remains uncertain.

## Conclusions

In disseminated PSL, sequential therapy with gemcitabine-docetaxel followed by pazopanib was feasible in this patient and was associated with prolonged disease control. Given the extreme rarity of PSL and the absence of standardized response assessment in this case, this observation should be interpreted as hypothesis-generating rather than practice-defining. Additional well-documented case reports and broader molecular characterization, when feasible, may help refine future management.
